# Understanding the Association Between Substance Use and Loneliness in Midlife and Older Adults

**DOI:** 10.3390/ijerph23020151

**Published:** 2026-01-26

**Authors:** Hermine Poghosyan, Jennifer McIntosh, Sayantani Sarkar, S. Raquel Ramos, Ophelia Empleo-Frazier, Nicole Colline, Shelli L. Feder

**Affiliations:** 1 Yale School of Nursing, Yale University, 400 West Campus Drive, Orange, CT 06477, USA; jennifer.mcintosh@yale.edu (J.M.); raquel.ramos@yale.edu (S.R.R.); ophelia.empleo-frazier@yale.edu (O.E.-F.); nicole.colline@yale.edu (N.C.); shelli.feder@yale.edu (S.L.F.); 2 Center for Information Technology Research in the Interest of Society (CITRIS), University of California, Berkeley, CA 94720, USA; sayantani.sarkar@berkeley.edu

**Keywords:** substance use, loneliness, midlife adults, older adults

## Abstract

**Highlights:**

**Public health relevance—How does this work relate to a public health issue?**

- Loneliness is a growing public health concern and is highly prevalent among adults aged ≥50 years.
- Substance use is a significant public health burden, as it places individuals at increased risk for morbidity and mortality.

**Public health significance—Why is this work of significance to public health?**

- Substance use and loneliness are increasing among adults aged ≥50 years, posing a growing public health burden.
- Understanding the link between substance use and loneliness can inform targeted prevention and early-intervention strategies for adults aged ≥50 years.

**Public health implications—What are the key implications or messages for practitioners, policy makers and/or researchers in public health?**

- Healthcare providers should routinely screen for both substance use and loneliness in adults aged ≥50 years.
- Age-specific, targeted strategies are urgently needed to reduce the burden of substance use and loneliness among adult populations.

**Abstract:**

Substance use, a significant public health concern, may be associated with worsening social connections and feelings of loneliness among adult populations. This study examined the associations between substance use (i.e., binge alcohol, e-cigarette, and traditional cigarette use) and loneliness among adults aged ≥50 years residing in the US. We conducted a secondary analysis of cross-sectional data from the 2023 Behavioral Risk Factor Surveillance System Social Determinant of Health Equity module. The sample included 138,614 adults aged ≥50 years, representing approximately 55.4 million individuals in this age group. Substance use was the key independent variable and categorized into two groups: no substance use and substance use—participants who reported current use of ≥1 of three substances (i.e., binge alcohol, e-cigarette, and traditional cigarette use). Overall, 19.0% of participants reported using ≥1 substance use, 21.0% reported feeling lonely sometimes, and 5.1% feeling lonely always/usually. Participants who reported using ≥1 substance had a 17.0% higher relative risk of feeling lonely always/usually (compared to feeling never/rarely lonely) than adults who did not report substance use, after adjusting for all covariates (RRR 1.17; 95% CI 1.10–1.35; *p* = 0.029). These findings underscore the strong link between substance use and loneliness among midlife and older adults.

## 1. Introduction

Loneliness and social isolation have emerged as pressing public health crises due to their profound impact on mental, emotional, and physical well-being [1,2,3]. Loneliness is a complex emotional state characterized by feelings of isolation and disconnection with a perceived discrepancy between an individual’s desired and actual social connections [1,4]. Conversely, social isolation refers to the objective condition of having minimal social relationships, roles, and interactions [5,6]. According to the World Health Organization, approximately 16.0% of people, one in six, experience loneliness globally, including 11.8% of older adults [7].

In the U.S., approximately 50.0% of the population reports experiencing loneliness, including about 34.0% of adults aged ≥50 years [8]. Loneliness has been identified as a significant risk factor for various mental and physical health problems [1,9]. Research suggests that experiencing chronic loneliness can be as harmful to health as smoking 15 cigarettes per day [1,10]. In fact, when people experience loneliness, they have a 26% higher risk of premature death [11,12]. The prevalence of loneliness, its risk factors, and consequences differ between age groups [13,14]. Previous work has shown that loneliness follows a U-shaped distribution, with peaks in late adolescence, young adulthood, and older adults [14,15]. Older adults are particularly vulnerable due to loss of close relationships, role changes, cognitive and physical decline, and impaired health status [1,16]. Moreover, loneliness among this group is particularly concerning due to its strong association with poor physical and mental health outcomes, such as cardiovascular disease, depression, anxiety, and increased mortality risk [1,17].

Substance use may exacerbate feelings of loneliness by disrupting relationships, weakening social support, and reducing social engagement. The relationship between substance use and loneliness among midlife and older adults is complex and may be influenced by various life stages and transitions. Middle to late adulthood, spanning ages 40 to 65, is a critical period during which individuals often juggle multiple roles in family, community, and work [18]. This stage, at times referred to as the “sandwich generation,” may be marked by stressors related to intergenerational dynamics, such as parenting older children, supporting grandchildren, and caring for aging parents while navigating work demands. These experiences can create significant emotional, financial, and time management strains. Studies have shown increased rates of anxiety, depression, and serious psychological distress among this group, particularly among individuals of lower socioeconomic status, women, and those identifying as lesbian, gay, bisexual, and transgender LGBTQ [19,20]; rates of antidepressant use are also higher among midlife individuals [21]. It is possible that during this period, the pressures and demands may contribute to varying levels of substance use and loneliness as individuals strive to manage these multifaceted roles.

Post-retirement, typically ages 66 to 89, is often a time when individuals may focus more on personal goals related to social relationships, as they are relieved of work- and family-related responsibilities [22]. As individuals age, relationship satisfaction tends to increase, despite a tendency for social networks to decrease [23]. However, retirement may also have negative consequences for health and well-being, potentially contributing to substance use and feelings of loneliness.

Reaching very old age, defined as 90 years and older, shifts the focus towards maintaining physical and cognitive functioning and striving for independence [22]. This stage is marked by declining capacities, increased awareness of the aging process’s irreversibility, and changes in family dynamics [24]. The transition into very old age is also marked by the sensation of progressive social exclusion associated with the loss of contemporaries or a spouse, challenges in connecting with younger generations, and a lack of meaningful community relationships [24]. These factors can lead to heightened feelings of loneliness and may influence substance use patterns as individuals navigate the realities of advanced age.

Moreover, adults aged ≥50 years are generally more vulnerable to the adverse effects of substance use, as age-related physiological changes such as slower metabolism, increased sensitivity, and other aging processes may predispose them to adverse health consequences [25,26,27,28]. Although cigarette use has declined significantly over the past decades, approximately 12.0% of adults aged ≥50 years still report current cigarette use, posing ongoing health risks in this vulnerable group [29]. Furthermore, about 10% of this age group of adults report binge alcohol drinking [29].The use of substances is often employed as a mechanism to suppress the feelings of loneliness and associated emotional distress [30]. Existing evidence has also found that the perceived feelings of loneliness can be influenced by several socio-demographic factors, such as race or ethnicity, educational level, and healthcare access. Similarly, substance use behaviors also vary widely by racial identity, educational level, sexual and gender identity, and other socio-demographic factors [9,31,32,33,34]. While substance use is well studied in the general population [35], its association with loneliness, particularly among this growing population of adults aged ≥50 years, remains inadequately studied, leaving a critical gap in understanding this association in mid and later life. Thus, this study estimated the prevalence of substance use (i.e., binge alcohol, e-cigarette, and traditional cigarette use) and loneliness among adults aged ≥50 residing in the US. Additionally, it examined the associations between substance use and self-reported loneliness among this population. By exploring these relationships, this study contributes to the expanding body of literature on aging, emotional well-being, and behavioral health, to inform both clinical practice and public health policy.

## 2. Materials and Methods

### 2.1. Data Source

This study used secondary cross-sectional data from the 2023 Behavioral Risk Factor Surveillance System (BRFSS), Social Determinants and Health Equity (SDHE) optional module [36]. The BRFSS is a nationally recognized, population-based survey designed to collect data from noninstitutionalized adults aged ≥18 across the US. Administered annually, BRFSS employs a standardized questionnaire composed of three components: a core set of questions administered by all states, optional modules that cover specific public health topics are administered by some states and state-added questions tailored to local needs. The survey collects data on socio-demographics, health-related risk behaviors, chronic health conditions, perceived health status, and access to healthcare services, among others. The total sample size for the 2023 BRFSS was 433,323, and the response rate was 44.7% [36].

### 2.2. Study Population

**Figure 1:**
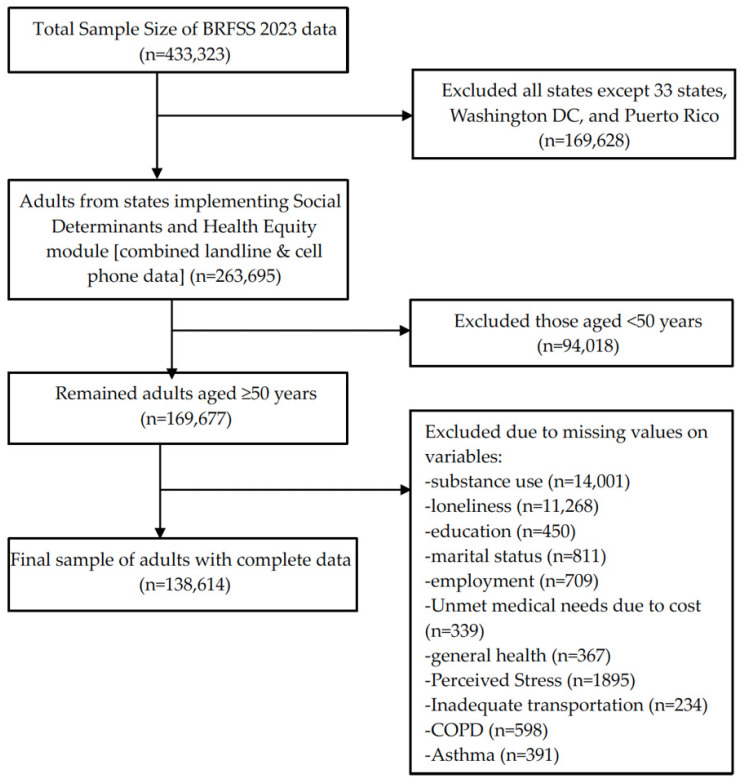
Flow chart displaying sample selection.

The data for the current study comes from the Districts of Columbia, 33 US states (Alabama, Alaska, Arizona, Arkansas, California, Connecticut, Delaware, Georgia, Hawaii, Idaho, Illinois, Indiana, Iowa, Kansas, Louisiana, Maine, Massachusetts, Minnesota, Mississippi, Missouri, Montana, Nevada, New Hampshire, New Jersey, New Mexico, North Carolina, North Dakota, Rhode Island, South Carolina, Utah, Virginia, West Virginia, Wisconsin) and one US territory (Puerto Rico) that administered the SDHE module in their 2023 BRFSS survey [36]. After excluding respondents from states that did not administer the SDHE module (*n* = 169,628) and individuals younger than 50 years (*n* = 94,018), the sample included 169,677 adults aged ≥50 years residing in selected US states and one territory. We further excluded observations with responses of “refused” or “don’t know/not sure,” or with missing values in the outcome, key predictor variables, or covariates. The final analytic sample included 138,614 adults aged ≥50 years. Figure 1 shows details of this study sample.

### 2.3. Study Variables

The primary outcome, loneliness, was measured using a single-item question from the SDHE module. Participants were asked, “How often do you feel lonely? with five response options: “always”, “usually”, “sometimes”, “rarely”, and “never”. This item measured the frequency of subjective feelings of loneliness. For the current study, responses were recoded into three categories reflecting increasing levels of loneliness frequency: (0) rarely/never, (1) sometimes, and (2) always/usually. This single-item measure of loneliness has also been used in previous research as a valid indicator of perceived loneliness [37,38].

The key predictor was self-reported substance use, which included current use of traditional cigarettes, electronic cigarettes, and binge alcohol drinking. Current traditional cigarette use was defined as having smoked at least 100 cigarettes in one’s lifetime and currently reported cigarette use every day or some days. Respondents who reported using at least 100 traditional cigarettes in their lifetime but reported no current use (former) and those who reported fewer than 100 traditional cigarettes in their entire lifetime (never) [39] are combined together for our study. This recategorization is done based on the prior published literature [40].

Current electronic cigarette use was defined as those reported using e-cigarettes every day or on some days, and individuals who reported never having used e-cigarettes and not currently using them were classified as the non-user group. Binge drinking was defined as consuming five or more alcohol drinks on a single occasion for males and four or more for females [41]. We combined these three behaviors into a binary substance use variable: (0) no substance use (participants who reported no current use of traditional cigarettes, e-cigarettes, or binge drinking), and (1) substance use (participants who reported current use of at least one of the three substances) [42].

We included a comprehensive set of covariates to adjust for potential confounding in the relationship between substance use and loneliness. Sociodemographic covariates included age (50–64, 65–74, 75–79, 80+), race and ethnicity (non-Hispanic Asian, non-Hispanic Black, Hispanic, non-Hispanic White, other race and ethnicity), sex (male, female) marital status (never married, divorced/separated, married or part of unmarried couple, widowed), education level (high school graduate or less, some college or technical school, college graduate), and employment status (employed, retired, not in a workforce). We also included variables for unmet medical needs and lack of reliable transportation. Unmet medical need (yes, no) was measured by asking participants the following question: “Was there a time in the past 12 months when you needed to see a doctor but could not because you could not afford it?” with yes/no responses. The lack of reliable transportation (yes, no) was assessed by the following question “During the past 12 months has a lack of reliable transportation kept you from medical appointments, meetings, work, or from getting things needed for daily living?”. Health-related covariates included self-reported general health status (fair/poor health, vs. good/very good/excellent health), history of asthma (yes, no), history of chronic obstructive pulmonary disease, emphysema, or chronic bronchitis (yes, no), and perceived stress. Perceived stress was measured with the following item “Stress means a situation in which a person feels tense, restless, nervous, or anxious, or is unable to sleep at night because his/her mind is troubled all the time. Within the last 30 days, how often have you felt this kind of stress?” Consistent with BRFSS, we recategorized it as a binary variable with the following categories: yes (always/usually) and no (rarely/never/sometimes) [43].

### 2.4. Statistical Analysis

We applied recommended sampling weights to account for the complex survey design of the BRFSS and to produce nationally representative population estimates. The BRFSS weighting process includes two key steps: (1) design weighting, which corrects for unequal probabilities of participant selection, and (2) iterative proportion fitting (ranking), which aligns the sample distribution with known population benchmarks. Ranking adjusts for potential nonresponse and sampling biases using demographic variables, including sex, age, race, Hispanic ethnicity, marital status, education, homeownership, phone ownership, and geographic region [36].

First, we conducted descriptive analyses to describe the study sample characteristics, presenting frequencies and weighted percentages along with their corresponding 95% confidence intervals (CI) for all study variables. We used chi-square tests, with Wald statistics, to estimate the prevalence of substance use and loneliness across sample characteristics. To examine the association between substance use and loneliness, we conducted a weighted multivariable multinomial logistic regression analysis. In this model, loneliness was treated as a three-category outcome variable, with ‘rarely/never’ serving as the reference category and comparisons made to both ‘sometimes’ and ‘always/usually’ categories. The model was adjusted for all covariates to control for potential confounding factors. Results are presented as Relative Risk Ratios (RRR) with 95% CIs for each independent variable. All analyses accounted for the complex survey design of the BRFSS using the ‘svy’ command in STATA 18 (StataCorp LLC, College Station, TX, USA). Statistical significance was assessed using two-tailed *p*-values, with a threshold of <0.05 considered statistically significant.

## 3. Results

### 3.1. Sample Characteristics

The sample included 138,614 adults aged ≥50 years, representing approximately 55.4 million individuals in this age group residing in the included US states and one territory. Sample characteristics are presented in Table 1. Overall, 67.3% of participants self-identified as non-Hispanic White, 12.8% as Hispanic, 10.9% as non-Hispanic Black, and 5.0% as Asian. Just over half (50.5%) were aged 50–64, and 54.0% were women. About 35.6% had high school or lower level of education, and 16.0% were not in the workforce. About 6.0% reported unmet medical needs due to cost, and 5.2% reported inadequate transportation in the past 12 months. Overall, 24.0% reported their general health as fair or poor, 9.0% reported experiencing perceived stress, 9.8% reported having lung disease, and 13.8% reported having asthma (Table 1).

### 3.2. Substance Use and Loneliness

Of study participants, 19.0% reported ≥1 substance use, with 10.9% reporting traditional cigarette use, 8.9% binge drinking, and 2.1% e-cigarette use. Overall, 21.0% reported feeling lonely sometimes, and 5.1% reported feeling lonely always/usually (Table 2). 

Table 3 presents the prevalence of loneliness by sample characteristics. Felling lonely always/usually was most prevalent among the younger group (aged 50–64 years), Hispanic individuals, divorced adults, those with a high school education or less, not in the workforce, those who experienced medical unmet need, had a lack of reliable transportation, and reported perceived stress. 

Table 4 presents the prevalence of substance use by sample characteristics. Among those who reported the use of ≥1 substance, 8.0% reported feeling lonely always/usually, and 24.0% feeling lonely sometimes, compared to 4.4% and 20.2% among nonusers, respectively. Substance use was more prevalent among younger adults (aged 50–64 years), non-Hispanic white individuals, men, divorced/separated individuals, and those with a lower education level.

### 3.3. Association Between Substance Use and Loneliness

Table 5 presents results from the adjusted multivariable multinomial logistic regression analysis. Adults aged ≥50 years who reported the use of ≥1 substance had 17.0% higher relative risk of reporting feeling lonely always/usually (compared to feeling never/rarely lonely) than adults who did not report substance use, after adjusting for all covariates (RRR 1.17; 95% CI 1.10–1.35; *p* = 0.029). Similarly, adults who reported ≥1 substance use had 10.0% higher risk of reporting feeling lonely sometimes (compared to feeling lonely never/rarely) than those not using substances, controlled for all covariates (RRR 1.10; 95% CI 1.01–1.20; *p* = 0.021).

## 4. Discussion

Our study findings demonstrated that substance use was associated with loneliness among midlife and older adults aged ≥50 years. These findings are consistent with prior literature suggesting that individuals who use substances such as alcohol and cannabis are more likely to report feeling lonely, compared to those who do not use such substances [44,45]. A study conducted with students found that substance use behaviors, such as binge drinking, nicotine vaping, and cannabis use, may significantly increase the risk of loneliness [46]. Another study showed that cigarette smoking can heighten loneliness [47]. These findings underscore the importance of substance use as a correlate of loneliness, warranting further investigation in the midlife and older adult population.

In our study of adults aged ≥50 years, we found that the prevalence of loneliness (sometimes/usually/always) was approximately 26%. A very recent study published with a much older age group (≥65) found a comparatively lower prevalence of loneliness (11.6%) [48]. These differing findings may be due to age differences and life stages of individuals in the study samples, particularly the inclusion of a comparatively younger age group (50–64 years old) in our sample. It is essential to note that these results may also be influenced by the life stages and transitions experienced by this age group. For instance, individuals at this stage often navigate multiple roles across family, community, and work [18]. This period may be characterized by the stressors of being in a “sandwich generation” with intergenerational dynamics, such as parenting older children, supporting grandchildren, and caring for aging parents, all while managing the demands of employment. Additionally, individuals in this cohort may be preparing for retirement, experiencing widowhood, and facing the changes associated with their children moving out, leading to “empty nest” situations.

Moreover, the literature suggests that loneliness is significantly associated with various negative health outcomes and medication use among older adults [11,49]. For instance, individuals who felt lonely were more likely to use antidepressants (27.0%) and benzodiazepines (11.0%) [49]. Furthermore, when individuals experience loneliness, they have a 26% higher risk for premature death [11]. In aggregate, these findings highlight the importance of comprehensive approaches to support the health and well-being of these individuals. Healthcare providers should routinely screen this group of individuals for loneliness, in addition to screening for substance use. Our findings suggested that substance use remains a significant concern in this aging population, with 19.0% of participants reporting the use of ≥1 substance. This is alarming as substance use places these individuals at increased risk for morbidity and mortality, while also complicating the management of chronic diseases that are highly prevalent in this age group such as cardiovascular disease, cancer, and diabetes [50,51,52]. Implementing tailored intervention programs to effectively reduce the burden of substance use among adult populations is urgently needed.

Our study findings showed that among the sample, some groups were more likely to report loneliness than others. For example, among racial and ethnic groups, loneliness was most prevalent among Hispanic individuals compared to their non-Hispanic white counterparts. A study conducted by Tibirica and colleagues (2022) investigated loneliness among Hispanic/Latinx men aged 50–68 years in the US and similarly reported higher levels of loneliness in this group compared to non-Hispanic individuals [53]. One potential explanation might be differences in social and structural factors that may contribute to increased loneliness among Hispanic individuals, such as language barriers, under-resourced community dwellings, and a lack of social support, underscoring the need for culturally responsive strategies and interventions to address loneliness. We also found that women were less likely than men to experience loneliness. However, evidence regarding sex differences in loneliness presents mixed results. Nicolaisen & Thorsen (2024) examined gender differences in loneliness over a 15-year time frame and found that older women, aged 60–80 years, experienced elevated levels of loneliness compared to men [54]. In contrast, a meta-analysis conducted by Maes et al. (2019) reported that men were slightly lonelier than women [55]. Similar findings have been observed in another study [56]. Future research is needed to clarify gender and sex differences associated with loneliness among midlife and older adults.

Several psychosocial and structural factors were also significantly associated with loneliness among our sample. Participants who experienced perceived stress during the past 30 days had 182% increased risk of feeling lonely. Stress, characterized by feelings of tension, anxiety, and restlessness, often accompanied by sleep disturbances, has been shown to intensify loneliness and may also increase vulnerability to substance use [57,58]. Those in the sample who lacked reliable transportation, which kept them from going to medical appointments, meetings, or work, had a 134% increased risk of reporting feeling lonely usually/always. Research conducted by Henning-Smith (2020) revealed that inadequate access to affordable and reliable transportation limits older adults’ ability to engage in community activities, attend medical appointments, and maintain social ties [59,60]. Our study findings highlight the importance of implementing interventions that address social determinants of health, demographic factors, and routine screening for substance use and loneliness among adult populations.

A notable strength of this study was the use of large nationally representative population-based data drawn from the 33 US states and one US territory that have contributed to the SDHE optional module. The relationship between loneliness and substance use is nuanced for aging populations. However, this study is not without limitations. Despite the large sample size, this study may not be generalizable to all US adults aged ≥50 years, as it did not include samples from all states, nor did all states administer the SDHE optional module of the BRFSS. All variables included in the study were self-reported and thus are at risk of response, recall, and social desirability bias. The cross-sectional design of the current study prevents establishing the causal relationship between substance use and loneliness. The temporality of the analysis may not adequately capture social issues affecting the aging population. Unequal group sizes for the substance use variable may have reduced statistical power and increased the risk of Type II error. Therefore, these findings should be interpreted with caution, and future research should aim for more balanced group sizes to facilitate replication. Loneliness in this study is measured with a single-item question that reflects its frequency, not its magnitude or severity. In addition, loneliness is a complex, self-perceived feeling that may arise due to multiple factors or a combination of such factors, such as limited social network, lack of closeness or poor relationship quality, lack of sense of belongingness in the social network, living arrangements, poor self-perceived health, and mental conditions such as depression [61]. Our study also did not include variables that may influence self-reported loneliness, such as an individual’s social network. Furthermore, our study has also investigated substance use as a binary variable, limiting the frequency, type, and extent of substance use that may influence the perception of loneliness. Future investigations should include a more thorough set of variables and validated loneliness scales to investigate the relationship between substance use and loneliness. Understanding contributing factors of loneliness in this growing population can guide primary prevention strategies and early treatment plans, delay long-term health effects, and thus decrease morbidity and mortality associated with loneliness.

## 5. Conclusions

Substance use is common among midlife and older adults and is associated with a higher likelihood of loneliness. Study findings highlight the emergent need for healthcare providers to integrate substance use and loneliness screening into routine clinical practice, particularly among adults aged ≥50. Our findings also emphasize the need to implement evidence-based interventions that address loneliness to mitigate the adverse effects of substance use and loneliness.

## Figures and Tables

**Table 1:** Characteristics of adults aged ≥50 years, BRFSS 2023.

Characteristics	Unweighted Frequences	Weighted Prevalence (95% CI)
	N = 138,614	Weighted n = 55,443,565
Age		
50–64	50,354	50.5 (49.9, 51.1)
65–74	46,803	29.1 (28.6, 29.6)
75–79	17,849	9.8 (9.5, 10.1)
≥80	19,608	10.6 (10.3, 11)
Sex		
Male	61,340	46.0 (45.4, 46.6)
Female	77,274	54.0 (53.4, 54.6)
Race and Ethnicity		
Non-Hispanic Asian	2467	5.0 (4.4, 5.5)
Non-Hispanic Black	9449	10.9 (10.5, 11.3)
Hispanic	8566	12.8 (12.4, 13.3)
Non-Hispanic White	112,380	67.3 (66.6, 67.9)
Other	5752	4.0 (3.8, 4.3)
Marital Status		
Never married	11,065	7.8 (7.5, 8.1)
Married/Unmarried Couple	80,911	62.8 (62.2, 63.4)
Divorced/separated	23,248	15.5 (15.1, 15.9)
Widowed	23,390	13.9 (13.5, 14.3)
Education		
Graduated college/technical school	60,689	32.9 (32.4, 33.5)
Attended college/technical school	38,394	31.4 (30.9, 32)
High-school or less	39,531	35.7 (35.1, 36.3)
Employment		
Employed	48,758	40.5 (39.9, 41.1)
Not in the workforce	17,814	15.9 (15.5, 16.4)
Retired	72,042	43.6 (43, 44.1)
Unmet medical need		
Yes	6800	6.0 (5.7, 6.2)
No	131,814	94.0 (93.8, 94.3)
Lack of reliable transportation		
Yes	6724	5.2 (4.9, 5.4)
No	131,890	94.8 (94.6, 95.1)
General health		
Good/better	108,524	76.1 (75.6, 76.6)
Fair/poor	30,090	23.9 (23.4, 24.5)
Perceived Stress		
No	127,580	91.0 (90.7, 91.4)
Yes	11,034	9.0 (8.6, 9.3)
Lung disease		
Yes	14,296	9.8 (9.5, 10.2)
No	124,318	90.2 (89.9, 90.5)
Asthma		
Yes	19,098	13.8 (13.4, 14.2)
No	119,516	86.2 (85.8, 86.6)

**Table 2:** Substance use and loneliness among adults aged ≥50 years.

Characteristics	Unweighted Frequences	Weighted Prevalence (95% CI)
**Smoking Status**		
Never/former	124,636	89.1 (88.7, 89.5)
Current	13,978	10.9 (10.5, 11.3)
**Binge Drinker**		
No	127,407	91.1 (90.8, 91.5)
Yes	11,207	8.9 (8.5, 9.2)
**Current E-Cigarette Use**		
No	135,959	97.9 (97.8, 98.1)
Yes	2655	2.1 (1.9, 2.2)
**Substance use**		
No use	114,196	81.0 (80.5, 81.4)
≥1 use	24,418	19.1 (18.6, 19.5)
**Loneliness**		
Rarely/never	103,305	74.0 (73.43, 74.5)
Sometimes	28,758	21.0 (20.5, 21.5)
Always/usually	6551	5.1 (4.8, 5.3)

**Table 3:** Prevalence of loneliness among adults aged ≥50 years by individual-level characteristics.

	Loneliness			
Characteristics	Rarely/Never	Sometimes	Always/Usually	*p*-Value
	Weighted Prevalence (95% CI)			
**Age**				<0.001
50–64	72.4 (71.6–73.3)	21.6 (20.9–22.4)	6.0 (5.5–6.4)	
65–74	75.7 (74.7–76.6)	20.2 (19.3–21.2)	4.1 (3.7–4.6)	
75–79	76.9 (75.5–78.2)	19.1 (17.9–20.4)	4.0 (3.4–4.7)	
≥80	74.1 (72.7–75.4)	21.6 (20.4–23.0)	4.3 (3.7–4.9)	
**Sex**				<0.001
Male	77.2 (76.5–78)	17.8 (17.1–18.5)	5.0 (4.6–5.4)	
Female	71.2 (70.4–72.0)	23.7 (23.0–24.4)	5.1 (4.8–5.5)	
**Race and Ethnicity**				<0.001
Non-Hispanic Asian	73.3 (68.1–78.0)	23.1 (18.7–28.2)	3.6 (2.0–6.2)	
Non-Hispanic Black	68.3 (66.6–70)	26.4 (24.8–28.0)	5.3 (4.5–6.3)	
Hispanic	68.2 (66.2–70.1)	23.1 (21.4–25.0)	8.7 (7.7–9.9)	
Non-Hispanic White	76.3 (75.8–76.8)	19.4 (18.9–19.9)	4.3 (4.1–4.6)	
Other	70.2 (67.1–73.1)	23.4 (20.7–26.4)	6.4 (5.1–8.0)	
**Marital Status**				
Never married	62.1 (60.0–64.1)	29.5 (27.6–31.5)	8.4 (7.5–9.5)	<0.001
Married/Unmarried Couple	81.8 (81.2–82.5)	15.4 (14.8–16.0)	2.8 (2.5–3.1)	
Divorced/separated	61.4 (60.0–62.7)	29.0 (27.7–30.3)	9.6 (8.0–10.5)	
Widowed	59.2 (57.7–60.6)	32.5 (31.1–34.0)	8.3 (7.6–9.2)	
**Education**				<0.001
Graduated college/technical school	79.0 (78.1–79.8)	18.0 (17.3–18.9)	3.0 (2.6–3.3)	
Attended college/technical school	74.1 (73.2–75.1)	21.2 (20.3–22.2)	4.7 (4.3–5.1)	
High-school or less	69.2 (68.2–70.2)	23.5 (22.6–24.3)	7.3 (6.8–7.9)	
**Employment**				<0.001
Employed	78.0 (77.1–78.8)	18.4 (17.7–19.2)	3.6 (3.2–4.0)	
Not in the workforce	57.9 (56.3–59.5)	30.1 (28.6–31.6)	12.0 (11.1–13.0)	
Retired	76.1 (75.4–76.9)	20.0 (19.3–20.7)	3.9 (3.6–4.3)	
**Unmet medical need**				<0.001
Yes	50.5 (48.0–53.0)	33.6 (31.2–36.1)	15.9 (14.2–17.6)	
No	75.4 (74.9–76.0)	20.2 (19.7–20.7)	4.4 (4.1–4.6)	
**Lack of reliable transportation**				<0.001
Yes	43.4 (40.9–45.9)	35.8 (33.2–38.5)	20.8 (19.8–22.9)	
No	75.7 (75.1–76.2)	20.1 (19.6–20.7)	4.2 (4.0–4.5)	
**General health**				<0.001
Good/better	78.7 (78.1–79.3)	18.2 (17.6–18.8)	3.1 (2.8–3.3)	
Fair/poor	58.8 (57.6–60.1)	29.8 (28.6–30.9)	11.4 (10.7–12.2)	
**Perceived Stress**				<0.001
Yes	38.2 (36.4–40.1)	36.5 (34.6–38.4)	25.3 (23.6–27.1)	
No	77.5 (77.0–78.0)	19.4 (18.9–20.0)	3.1 (2.9–3.3)	
**Lung disease**				<0.001
Yes	60.9 (59.2–62.7)	27.7 (26.1–29.3)	11.4 (10.3–12.6)	
No	75.4 (74.8–76.0)	20.2 (19.7–20.8)	4.4 (4.1–4.6)	
**Asthma**				<0.001
Yes	65.5 (63.9–67.0)	26.1 (24.7–27.6)	8.4 (7.6–9.3)	
No	75.3 (74.8–75.9)	20.1 (19.6–20.7)	4.5 (4.3–4.8)	
**Substance use**				<0.001
No	75.4 (74.8–76.0)	20.2 (19.7–20.8)	4.4 (4.1–4.7)	
≥1 use	68.0 (66.6–69.3)	24.0 (22.8–25.3)	8.0 (7.3–8.7)	

**Table 4:** Prevalence of substance use among adults aged ≥50 years by individual-level characteristics.

	Substance Use		
Characteristics	No	≥1 Use	*p*-Value
	Weighted Prevalence (95% CI)		
**Age**			<0.001
50–64	74.8 (74.1–75.6)	25.2 (24.4–26.0)	
65–74	83.6 (82.8–84.3)	16.4 (15.7–17.2)	
75–79	89.8 (88.8–90.8)	10.2 (9.2–11.2)	
≥80	94.7 (94.1–95.3)	5.3 (4.7–5.9)	
**Sex**			<0.001
Male	77.1 (76.4–77.9)	22.9 (22.1–23.7)	
Female	84.2 (83.6–84. 8)	15.8 (15.2–16.4)	
**Race and Ethnicity**			<0.001
Non-Hispanic Asian	91.2 (87.6–93.8)	8.8 (6.2–12.4)	
Non-Hispanic Black	81.9 (80.4–83.2)	18.1 (16.8–19.6)	
Hispanic	83.4 (81.7–85.0)	16.6 (15.0–18.3)	
Non-Hispanic White	79.9 (79.4–80.4)	20.1 (19.6–20.6)	
Other	75.4 (72.5–78.0)	24.6 (22.0–27.5)	
**Marital Status**			<0.001
Never married	75.1 (73.0–77.0)	24.9 (23.0–27.0)	
Married/Unmarried Couple	83.2 (82.6–83.8)	16.8 (16.2–17.4)	
Divorced/separated	72.3 (71.1–73.5)	27.7 (26.5–29.0)	
Widowed	83.8 (82.5–85.1)	16.2 (14.9–17.5)	
**Education**			<0.001
Graduated college/technical school	87.6 (87.0–88.2)	12.4 (11.8–13.0)	
Attended college/technical school	79.5 (78.6–80.4)	20.5 (19.6–21.4)	
High-school or less	76.1 (75.1–77.0)	23.9 (23.1–24.9)	
**Employment**			<0.001
Employed	77.9 (77.1–78.7)	22.1 (21.3–22.9)	
Not in the workforce	74.8 (73.4–76.3)	25.2 (23.7–26.6)	
Retired	86.0 (85.4–86.6)	14.0 (13.4–14.6)	
**Unmet medical need**			<0.001
Yes	68.6 (66.1–70.9)	31.4 (29.1–33.8)	
No	81.7 (81.3–82.2)	18.3 (17.8–18.8)	
**Lack of reliable transportation**			<0.001
Yes	67.4 (64.8–69.8)	32.6 (30.2–35.2)	
No	81.7 (81.2–82.2)	18.3 (17.8–18.8)	
**General health**			<0.001
Good/better	82.1 (81.6–82.7)	17.9 (17.4–18.4)	
Fair/poor	77.3 (76.2–78.3)	22.7 (21.7–23.8)	
**Perceived Stress**			<0.001
Yes	68.3 (66.5–70.1)	31.7 (30.0–33.5)	
No	82.2 (81.7–82.7)	17.8 (17.3–18.3)	
**Lung disease**			<0.001
Yes	62.0 (60.3–63.8)	38.0 (36.2–39.7)	
No	83.0 (82.5–83.5)	17.0 (16.5–17.5)	
**Asthma**			0.5
Yes	80.6 (79.4–81.7)	19.4 (18.3–20.6)	
No	81.0 (80.5–81.5)	19.0 (18.5–19.5)	
**Loneliness**			<0.001
Rarely/never	82.5 (82.0–83)	17.5 (17.0–18.0)	
Sometimes	78.2 (77.0–79.0)	21.8 (20.7–23.0)	
Always/usually	70.0 (67.5–72.2)	30.0 (27.8–32.5)	

**Table 5:** Association between substance use and loneliness among adults aged ≥50 years.

Characteristics	Sometimes vs. Rarely/Never RRR (95% CI)	*p*-Value	Always/Usually vs. Rarely/Never RRR (95% CI)	*p*-Value
**Substance use**				
No use	1		1	
Yes, ≥1 use	1.10 (1.02–1.20)	0.021	1.17 (1.02–1.36)	0.029
**Age**				
50–64	1		1	
65–74	0.91 (0.83–1.00)	0.055	0.83 (0.68–1.00)	0.055
75–79	0.79 (0.70–0.89)	<0.001	0.78 (0.61–0.99)	0.043
≥80	0.78 (0.69–0.89)	<0.001	0.77 (0.61–0.98)	0.035
**Sex**				
Male	1		1	
Female	1.21 (1.13–1.29)	<0.001	0.80 (0.70–0.91)	0.001
**Race and Ethnicity**				
Non-Hispanic White	1		1	
Non-Hispanic Asian	1.59 (1.20–2.12)	0.001	1.59 (0.88–2.89)	0.125
Non-Hispanic Black	1.16 (1.06–1.28)	0.002	0.91 (0.73–1.13)	0.399
Hispanic	1.05 (0.93–1.17)	0.440	1.47 (1.23–1.76)	0.000
Other	1.05 (0.89–1.24)	0.568	0.96 (0.71–1.30)	0.804
**Marital Status**				
Married/Unmarried Couple	1		1	
Never married	2.11 (1.88–2.36)	<0.001	2.78 (2.27–3.40)	<0.001
Divorced/separated	2.12 (1.96–2.30)	<0.001	3.11 (2.66–3.64)	<0.001
Widowed	2.93 (2.66–3.22)	<0.001	4.43 (3.74–5.26)	<0.001
**Education**				
High-school or less	1		1	
Attended college/technical school	0.99 (0.92–1.08)	0.880	0.83 (0.72–0.95)	0.007
Graduated college/technical school	0.97 (0.90–1.05)	0.426	0.78 (0.67–0.91)	0.001
**Employment**				
Not in the workforce	1		1	
Employed	0.73 (0.66–0.82)	<0.001	0.58 (0.49–0.69)	<0.001
Retired	0.79 (0.70–0.88)	<0.001	0.67 (0.55–0.82)	<0.001
**Unmet medical need**				
No	1		1	
Yes	1.49 (1.31–1.70)	<0.001	1.79 (1.48–2.17)	<0.001
**Lack of reliable transportation**				
No	1		1	
Yes	1.56 (1.35–1.79)	<0.001	2.34 (1.88–2.92)	<0.001
**General health**				
Good/better	1		1	
Fair/poor	1.57 (1.45–1.69)	<0.001	2.08 (1.81–2.39)	<0.001
**Perceived Stress**				
No	1		1	
Yes	2.82 (2.54–3.13)	<0.001	9.94 (8.52–11.59)	<0.001
**Lung disease**				
No	1		1	
Yes	1.05 (0.95–1.16)	0.355	1.28 (1.07–1.41)	0.002
**Asthma**				
No	1		1	
Yes	1.18 (1.08–1.30)	<0.001	1.23 (1.07–1.41)	0.003

## Data Availability

The data used in this study are open and publicly available at https://www.cdc.gov/brfss/ (accessed on 9 September 2024).

## Abbreviations

The following abbreviations are used in this manuscript:

BRFSS	Behavioral Risk Factor Surveillance System
SDHE	Social Determinants and Health Equity

## References

[1] U.S. Department of Health and Human Services (2023). Our Epidemic of Loneliness and Isolation: The U.S. Surgeon General’s Advisory on the Healing Effects of Social Connection and Community. https://www.hhs.gov/sites/default/files/surgeon-general-social-connection-advisory.pdf.

[2] Zeas-Siguenza A., Ruisoto P., Koldewyn K., Muntane F., Benach J. (2025). Beyond clinical risk: Tackling loneliness through a population health lens. Front. Psychol..

[3] Zeas-Siguenza A., Voldstad A., Ruisoto P., Ganho-Avila A., Guiomar R., Cacho R., Muntane F., Benach J. (2025). Loneliness as a Public Health Challenge: A Systematic Review and Meta-Analysis to Inform Policy and Practice. Eur. J. Investig. Health Psychol. Educ..

[4] Prohaska T., Burholt V., Burns A., Golden J., Hawkley L., Lawlor B., Leavey G., Lubben J., Perissinotto C., van Tilburg T. (2020). Consensus statement: Loneliness in older adults, the 21st century social determinant of health?. BMJ Open.

[5] Badcock J., Holt-Lunstad J., Garcia E., Bombaci P., Lim M. (2022). Position Statement: Addressing Social Isolation and Loneliness and the Power of Human Connection.

[6] Holt-Lunstad J., Steptoe A. (2022). Social isolation: An underappreciated determinant of physical health. Curr. Opin. Psychol..

[7] World Health Organization [WHO] (2025). Reducing Social Isolation and Loneliness Among Older People. https://www.who.int/activities/reducing-social-isolation-and-loneliness-among-older-people.

[8] Malani P., Singer D., Kirch M., Solway E., Roberts S., Smith E., Hutchens L., Kullgren J. (2023). Trends in Loneliness Among Older Adults from 2018–2023 (University of Michigan National Poll on Healthy Aging, March 2023).

[9] National Academies of Sciences, Engineering, and Medicine (2020). Social Isolation and Loneliness in Older Adults: Opportunities for the Health Care System.

[10] Holt-Lunstad J., Smith T.B., Layton J.B. (2010). Social relationships and mortality risk: A meta-analytic review. PLoS Med..

[11] Holt-Lunstad J., Smith T.B., Baker M., Harris T., Stephenson D. (2015). Loneliness and social isolation as risk factors for mortality: A meta-analytic review. Perspect. Psychol. Sci..

[12] Smith R.W., Holt-Lunstad J., Kawachi I. (2023). Benchmarking Social Isolation, Loneliness, and Smoking: Challenges and Opportunities for Public Health. Am. J. Epidemiol..

[13] Carter B., Qualter P., Dix J. (2015). Social relationships, loneliness and adolescence: The potential for disruption by chronic illness. J Child Health Care.

[14] Qualter P., Vanhalst J., Harris R., Van Roekel E., Lodder G., Bangee M., Maes M., Verhagen M. (2015). Loneliness across the life span. Perspect. Psychol. Sci..

[15] Graham E.K., Beck E.D., Jackson K., Yoneda T., McGhee C., Pieramici L., Atherton O.E., Luo J., Willroth E.C., Steptoe A. (2024). Do we become more lonely with age? A coordinated data analysis of nine longitudinal studies. Psychol. Sci..

[16] Akhter-Khan S.C., Prina M., Wong G.H.-Y., Mayston R., Li L. (2023). Understanding and addressing older adults’ loneliness: The social relationship expectations framework. Perspect. Psychol. Sci..

[17] Mann F., Wang J., Pearce E., Ma R., Schlief M., Lloyd-Evans B., Ikhtabi S., Johnson S. (2022). Loneliness and the onset of new mental health problems in the general population. Soc. Psychiatry Psychiatr. Epidemiol..

[18] Infurna F.J., Gerstorf D., Lachman M.E. (2020). Midlife in the 2020s: Opportunities and challenges. Am. Psychol..

[19] Brody D.J., Pratt L.A., Hughes J.P. (2018). Prevalence of Depression Among Adults Aged 20 and Over: United States, 2013–2016. NCHS Data Brief.

[20] Fredriksen-Goldsen K.I., Jen S., Bryan A.E.B., Goldsen J. (2018). Cognitive Impairment, Alzheimer’s Disease, and Other Dementias in the Lives of Lesbian, Gay, Bisexual and Transgender (LGBT) Older Adults and Their Caregivers: Needs and Competencies. J. Appl. Gerontol..

[21] Blanchflower D.G., Oswald A.J. (2016). Antidepressants and age: A new form of evidence for U-shaped well-being through life. J. Econ. Behav. Organ..

[22] Freund A.M. (2020). The bucket list effect: Why leisure goals are often deferred until retirement. Am. Psychol..

[23] Buhler J.L., Nikitin J. (2020). Sociohistorical context and adult social development: New directions for 21st century research. Am. Psychol..

[24] Escourrou E., Laurent S., Leroux J., Oustric S., Gardette V. (2022). The shift from old age to very old age: An analysis of the perception of aging among older people. BMC Prim. Care.

[25] Dowling G.J., Weiss S.R., Condon T.P. (2008). Drugs of abuse and the aging brain. Neuropsychopharmacology.

[26] Gossop M., Moos R. (2008). Substance misuse among older adults: A neglected but treatable problem. Addiction.

[27] Hanson G.L., Venturelli P.J., Fleckenstein A.E. (2018). Drugs and Society.

[28] Wu L.T., Blazer D.G. (2011). Illicit and nonmedical drug use among older adults: A review. J. Aging Health.

[29] You E., Sarkar S., Pietrzak R.H., Monin J.K., Poghosyan H. (2025). How Cannabis Use is Associated with the Physical and Mental Health of Older Adults: A US Population-Based Study. Int. J. Ment. Health Addict..

[30] Gutkind S., Gorfinkel L.R., Hasin D.S. (2022). Prospective effects of loneliness on frequency of alcohol and marijuana use. Addict. Behav..

[31] Fierloos I.N., Tan S.S., Williams G., Alhambra-Borras T., Koppelaar E., Bilajac L., Verma A., Markaki A., Mattace-Raso F., Vasiljev V. (2021). Socio-demographic characteristics associated with emotional and social loneliness among older adults. BMC Geriatr..

[32] Lam J., Campbell A. (2022). Trajectories of Loneliness Among Older Women and Men: Variation by Sexual Identity?. Gerentologist.

[33] Meza R., Cao P., Jeon J., Warner K.E., Levy D.T. (2023). Trends in US Adult Smoking Prevalence, 2011 to 2022. JAMA Health Forum.

[34] Napoles A.M., Stewart A.L., Strassle P.D., Alhomsi A., Quintero S., Ponce S., Wilkerson M., Bonilla J. (2023). Depression Symptoms, Perceived Stress, and Loneliness During the COVID-19 Pandemic Among Diverse US Racial-Ethnic Groups. Health Equity.

[35] Lu W., Lopez-Castro T., Vu T. (2023). Population-based examination of substance use disorders and treatment use among US young adults in the National Survey on drug use and health, 2011–2019. Drug Alcohol Depend. Rep..

[36] Centers for Disease Control and Prevention (2023). Behavioral Risk Factor Surveillance System (2023) BRFSS Survey Data and Documentation. https://www.cdc.gov/brfss/annual_data/annual_2023.html.

[37] Akinyemi O., Abdulrazaq W., Fasokun M., Ogunyankin F., Ikugbayigbe S., Nwosu U., Michael M., Hughes K., Ogundare T. (2025). The impact of loneliness on depression, mental health, and physical well-being. PLoS ONE.

[38] Falk D.S., Melgoza E., Cabrera A., Vazquez C.E. (2025). Loneliness in adults with cardiovascular disease and their social and emotional support needs: Implications for Hispanic adults from the 2023 Behavioral Risk Factor Surveillance System. medRxiv.

[39] Centers for Disease Control and Prevention (2025). Glossary. https://archive.cdc.gov/www_cdc_gov/nchs/nhis/tobacco/tobacco_glossary.htm.

[40] Mevawalla A., Khalil M., Rashid Z., Altaf A., Sarfraz A., Pawlik T.M. (2025). Cancer screening adherence among e-cigarette users in the United States. Tob. Prev. Cessat..

[41] National Institute on Alcohol Abuse and Alcoholism (2025). Understanding Binge Drinking. https://www.niaaa.nih.gov/publications/brochures-and-fact-sheets/binge-drinking.

[42] Sarkar S., Jackson B., Manzo L.L., Jeon S., Poghosyan H. (2024). Association between adverse childhood experiences and self-reported health-risk behaviors among cancer survivors: A population-based study. PLoS ONE.

[43] Hacker K., Thomas C.W., Zhao G., Claxton J.S., Eke P., Town M. (2024). Social Determinants of Health and Health-Related Social Needs Among Adults with Chronic Diseases in the United States, Behavioral Risk Factor Surveillance System, 2022. Prev. Chronic Dis..

[44] Bonar E.E., Walton M.A., Carter P.M., Lin L.A., Coughlin L.N., Goldstick J.E. (2022). Longitudinal within- and between-person associations of substance use, social influences, and loneliness among adolescents and emerging adults who use drugs. Addict. Res. Theory.

[45] Ingram I., Kelly P.J., Deane F.P., Baker A.L., Goh M.C.W., Raftery D.K., Dingle G.A. (2020). Loneliness among people with substance use problems: A narrative systematic review. Drug Alcohol Rev..

[46] Barry C.M., Jagtiani A., Skinner J.R., Gassaway A.N., Komro K.A., Livingston M.D. (2025). Loneliness among emerging adults in rural reservation-based communities: Longitudinal effects of 12th grade substance use and mental health symptoms. Sci. Rep..

[47] Wootton R.E., Greenstone H.S.R., Abdellaoui A., Denys D., Verweij K.J.H., Munafo M.R., Treur J.L. (2021). Bidirectional effects between loneliness, smoking and alcohol use: Evidence from a Mendelian randomization study. Addiction.

[48] Pollak C., Pham Y., Ehrlich A., Verghese J., Blumen H.M. (2025). Loneliness and social isolation risk factors in community-dwelling older adults receiving home health services. BMC Geriatr..

[49] Kotwal A.A., Steinman M.A., Cenzer I., Smith A.K. (2021). Use of high-risk medications among lonely older adults: Results from a nationally representative sample. JAMA Intern. Med..

[50] AARP (2022). Chronic Care: A Call to Action for Health Reform. Chronic Conditions Among Older Americans. https://assets.aarp.org/rgcenter/health/beyond_50_hcr_conditions.pdf.

[51] Atella V., Piano Mortari A., Kopinska J., Belotti F., Lapi F., Cricelli C., Fontana L. (2019). Trends in age-related disease burden and healthcare utilization. Aging Cell.

[52] McGrath R., Al Snih S., Markides K., Hall O., Peterson M. (2019). The burden of health conditions for middle-aged and older adults in the United States: Disability-adjusted life years. BMC Geriatr..

[53] Tibirica L., Jester D.J., Jeste D.V. (2022). A systematic review of loneliness and social isolation among Hispanic/Latinx older adults in the United States. Psychiatry Res..

[54] Nicolaisen M., Thorsen K. (2024). Gender differences in loneliness over time: A 15-year longitudinal study of men and women in the second part of life. Int. J. Aging Hum. Dev..

[55] Maes M., Qualter P., Vanhalst J., Van den Noortgate W., Goossens L. (2019). Gender Differences in Loneliness across the Lifespan: A Meta–Analysis. Eur. J. Personal..

[56] Barreto M., Victor C., Hammond C., Eccles A., Richins M.T., Qualter P. (2021). Loneliness around the world: Age, gender, and cultural differences in loneliness. Personal. Individ. Differ..

[57] Qi X., Malone S.K., Pei Y., Zhu Z., Wu B. (2023). Associations of social isolation and loneliness with the onset of insomnia symptoms among middle-aged and older adults in the United States: A population-based cohort study. Psychiatry Res..

[58] Segrin C., McNelis M., Pavlich C.A. (2018). Indirect Effects of Loneliness on Substance Use through Stress. Health Commun..

[59] Henning-Smith C. (2020). The Public Health Case for Addressing Transportation-Related Barriers to Care. Am. J. Public Health.

[60] Henning-Smith C. (2020). Strategies for Promoting Successful Aging and Well-Being. J. Appl. Gerontol..

[61] Dahlberg L., McKee K.J., Frank A., Naseer M. (2022). A systematic review of longitudinal risk factors for loneliness in older adults. Aging Ment. Health.

